# Insights into Inflammatory Priming of Adipose-Derived Mesenchymal Stem Cells: Validation of Extracellular Vesicles-Embedded miRNA Reference Genes as A Crucial Step for Donor Selection

**DOI:** 10.3390/cells8040369

**Published:** 2019-04-23

**Authors:** Enrico Ragni, Paola De Luca, Carlotta Perucca Orfei, Alessandra Colombini, Marco Viganò, Gaia Lugano, Valentina Bollati, Laura de Girolamo

**Affiliations:** 1 Laboratorio di Biotecnologie Applicate all’Ortopedia, IRCCS Istituto Ortopedico Galeazzi, I-20161 Milan, Italy; deluca.paola@grupposandonato.it (P.D.L.); carlotta.perucca@grupposandonato.it (C.P.O.); alessandra.colombini@grupposandonato.it (A.C.); marco.vigano@grupposandonato.it (M.V.); gaia.lugano@grupposandonato.it (G.L.); laura.degirolamo@grupposandonato.it (L.d.G.); 2 EPIGET—Epidemiology, Epigenetics and Toxicology Lab, Department of Clinical Sciences and Community Health, University of Milan, I-20122 Milan, Italy; valentina.bollati@unimi.it

**Keywords:** adipose-mesenchymal stem cells, extracellular vesicles, osteoarthritis, miRNA, reference gene, inflammation

## Abstract

Mesenchymal stem cells (MSCs) are promising tools for cell-based therapies due to their homing to injury sites, where they secrete bioactive factors such as cytokines, lipids, and nucleic acids, either free or conveyed within extracellular vesicles (EVs). Depending on the local environment, MSCs’ therapeutic value may be modulated, determining their fate and cell behavior. Inflammatory signals may induce critical changes on both the phenotype and secretory portfolio. Intriguingly, in animal models resembling joint diseases as osteoarthritis (OA), inflammatory priming enhanced the healing capacity of MSC-derived EVs. In this work, we selected miRNA reference genes (RGs) from the literature (let-7a-5p, miR-16-5p, miR-23a-3p, miR-26a-5p, miR-101-3p, miR-103a-3p, miR-221-3p, miR-423-5p, miR-425-5p, U6 snRNA), using EVs isolated from adipose-derived MSCs (ASCs) primed with IFNγ (iASCs). geNorm, NormFinder, BestKeeper, and ΔCt methods identified miR-26a-5p/16-5p as the most stable, while miR-103a-rp/425-5p performed poorly. Our results were validated on miRNAs involved in OA cartilage trophism. Only a proper normalization strategy reliably identified the differences between donors, a critical factor to empower the therapeutic value of future off-the-shelf MSC-EV isolates. In conclusion, the proposed pipeline increases the accuracy of MSC-EVs embedded miRNAs assessment, and help predicting donor variability for precision medicine approaches.

## 1. Introduction

Cell-based therapies are one of the most promising approaches in the medical arsenal to restore the lost function rather than producing new organs or tissue. The predicted target diseases are broad, such as hormonal dysfunction (diabetes and growth hormones); neurodegeneration (Parkinson, Alzheimer and Huntington); cardiovascular lesions (infarction and ischemia); and orthopedic such as skeletal muscle, joints, and bones (such as osteoarthritis-OA) [1]. Several resources of cells can be used to renovate the damaged tissues, and human mesenchymal stromal cells (MSCs) have been identified as a leading candidate [2]. MSCs may be easily isolated from almost all stromal tissues, have potential for ex vivo expansion and the ability to secrete a range of trophic factors, either free (cytokines or chemokines) or conveyed within extracellular vesicles (EVs) (proteins, lipids and nucleic acids as miRNAs) [3]. Due to these features, MSCs have been more recently defined as “Medicinal Signaling Cells”, since those molecules can orchestrate the trophism and regeneration of diseased tissues [4]. In this perspective, MSCs represent an innovation from current biopharmaceutical production, where the “medicine” of interest is not the cell itself but its released molecules [5]. This crucial difference greatly increases the grade of complexity in the field, taking into account both the manufacturing process and multiple donor variability.

One of most imperative aspects of manufacturing MSC-based therapeutic products is the definition and measurement of cell characteristics, which are greatly influenced by both donor selection and culturing conditions [6]. In the allogeneic settings, identifying the most appropriate batch depends on its fine characterization under different aspects, such as identity, potency, purity, and safety [7]. Beyond these more traditional indications, identifying donor-dependent levels of immunomodulatory and trophic factors (cytokines and EVs, in their whole defined as “secretome”) is a crucial aspect [8]. It is therefore important to understand how they will affect the final product’s identity and how the production process may alter these factors.

MSCs, and as consequence their secretome, can be modulated to boost the beneficial actions of the cells through priming or preconditioning. As an example, inflammatory priming induces cellular changes that drive cells and secreted factors towards a more anti-inflammatory phenotype, which can eventually lead to a more effective response against future severe inflammatory events [9]. Moreover, MSC inflammatory licensing was able to generate a more homogeneous population, compared to naïve MSCs, thereby improving the consistency of MSCs from different donors [10,11,12]. In the OA setting characterized by both tissue degeneration and inflammation, the conditioned media of activated MSCs showed enhanced chondroprotective effect [13,14]. Furthermore, primed MSCs exert a more pronounced immunosuppressive function [15] since their pre-activation in presence of interferon-γ (IFNγ) and/or tumor necrosis factor (TNF)-α, interleukin (IL1)-α, or IL1-β improves their anti-inflammatory effect [16]. In an in vivo mouse model of OA, inflammatory-primed MSC from adipose tissue (ASCs) prevented articular cartilage degradation—being more potent than naïve cells—an effect related to the modulation of their secretome [17]. Consistently, both conditioned medium and EVs obtained from IFNγ licensed-ASCs (iASCs) revealed a superior immunosuppressive potential compared to unlicensed cells [18].

These results have spurred the development of new approaches to characterize the molecular signature of licensed ASCs and their secreted factors to develop more efficacious clinical treatments, aimed at future personalized medicine strategies for individual analysis of the patient’s unique biology. In this frame, the identification of reliable reference genes (RGs) for correct quantification of iASC-EV-embedded miRNAs was performed and further validated on a panel of candidates involved in OA pathology to evaluate donor-dependent signatures, a fundamental step towards precision medicine approaches.

## 2. Materials and Methods

### 2.1. Ethics Statement

The research was conducted at IRCCS Istituto Ortopedico Galeazzi with Institutional Review Board approval and specimens were collected under patient informed consent (M-SPER-015-Ver. 2-04.11.2016), and following the 1964 Helsinki declaration and its later amendments or comparable ethical standards.

### 2.2. ASCs Isolation and Expansion

Waste material of five female donors (median 57 yo) undergoing elective plastic surgery (liposuction) was used to obtain adipose tissue samples that were digested for 30 min at 37 °C with 0.075% *w*/*v* type I collagenase (Worthington Biochemical Co, Lakewood, NJ, USA). After digestion, samples were filtered through a cell strainer and centrifuged (1000× *g*, 5 min) [19]. Cells were seeded at 5 × 10^3^ cells/cm^2^ in DMEM + 10% FBS (GE Healthcare, Piscataway, NJ, USA), L-glutamine and Penicillin-Streptomycin (Life Technology, Carlsbad, CA, USA). Cells were kept at 37 °C, 5% CO_2_, and 95% humidity. Experiments were performed with cells at passages 4 for flow cytometry and passage 5 for EVs collection. To prime ASCs with inflammation, 10 ng/mL IFNγ was added for 48 h.

### 2.3. ASC Characterization

Flow cytometry was used to score positive or negative MSC or hemato/endothelial markers (CD44-PE clone DB105, CD73-PE clone REA804, CD90-FITC clone REA897 and CD105-PerCP Vio700 clone REA794 or CD34-PE Vio770 clone AC136 and CD45-PE Vio770 clone REA747; Miltenyi Biotec, Bergisch Gladbach, Germany) [20,21] with a CytoFLEX flow cytometer (Beckman Coulter, Fullerton, CA, USA) collecting a minimum of 30,000 events [22]. Abs were used in the following combinations: CD73/90/105/45 and CD44/34.

### 2.4. iASC-EV Isolation and Characterization

After 48 h of IFNγ priming, cells were washed three times with PBS and DMEM without FBS added for another 48 h. Conditioned medium was collected and subjected to differential centrifugation steps to remove broken cells and debris, as described in [22]. Last supernatant was centrifuged at 100,000× *g* for 9 h at 4 °C in a 70Ti rotor (Beckman Coulter, Fullerton, CA, USA), and EV pellets were processed with the following different techniques.

Flow cytometry: EVs were suspended in 1 mL of Diluent C and 6 μL of PKH26 (Sigma-Aldrich, St. Louis, MO, USA) was added, and incubated for 5 min at RT. PKH26 was quenched with the addition of 2 mL of BSA at 100 mg per ml. A sucrose solution (1.5 mL of a 0.971 M) was added into the bottom of an ultracentrifuge tube, and the solution was centrifuged at 190,000× *g* at 4 °C for 2 h. Pellets containing labeled EVs were suspended in PBS and stained with anti CD81-FITC clone JS-81 (Becton Dickinson, NJ, USA), anti CD44-PE clone DB105 (for CFSE stained EVs as following described)/CD63-PE Vio770 clone H5C6/CD73-PE clone REA804 (for CFSE stained EVs)/CD90-FITC clone REA897/CD105-PerCP Vio700 clone REA794, and CD34-PE Vio770 clone AC136/CD45-PE Vio770 clone REA747 (Miltenyi Biotec, Bergisch Gladbach, Germany) for 30 min at 4 °C in the dark. Antibodies were used individually. Collection was performed with a CytoFLEX flow cytometer collecting a minimum of 30,000 events. To assess iASC-EV integrity, conditioned media were supplemented with 10 µM CFSE (Sigma-Aldrich) and incubated for 1 h at 37 °C. After ultracentrifugation, as previously described, pellets were suspended in PBS and vesicles analyzed with Cytoflex comparing outcomes with those obtained running FITC-fluorescent beads of 100, 300, 500, and 900 nm (Biocytex, Marseille, France).

Transmission electron microscopy: 5 µL of purified iASC-EVs in PBS after pellet suspension were absorbed on Formvar carbon-coated grids for 10 min. Filter paper was used to blot the drops. Negative staining was performed with a 2% uranyl acetate aqueous suspension for 10 min. The excess of uranyl was removed by filter paper by touching the grid that was dried at room temperature. Samples were examined with a TALOS L120C transmission electron microscope (Thermo Fisher Scientific, Waltham, MA, USA) at 120 kV.

Nanoparticle tracking analysis (NTA): iASC-EVs in suspension after ultracentrifugation were visualized by Nanosight LM10-HS system (NanoSight Ltd., Amesbury, UK). Three recordings of 30 s were performed for each EV sample. NTA software analyzed collected data, providing high-resolution particle size distribution profiles and concentration measurements.

### 2.5. Candidate RGs Selection

According to previously published papers scoring EV-miRNA RGs [23,24,25,26,27,28,29,30,31,32], one small RNA (U6 snRNA) and nine miRNAs (let-7a-5p, miR-16-5p, miR-23a-3p, miR-26a-5p, miR-101-3p, miR-103a-3p, miR-221-3p, miR-423-5p, and miR-425-5p) were selected.

### 2.6. Selection of OA-Related miRNAs

According to previously published data, 46 miRNAs related to cartilage homeostasis during OA were selected [33], considering only miRNAs with unambiguous effect (either considered as protective or destructive). Cartilage-protective: miR-320a-3p, miR-27b-3p, miR-148a-3p, miR-127-5p, miR-140-5p, miR-30a-5p, miR-92a-3p, miR-502-5p, miR-130a-3p, miR-193b-3p, miR-149-5p, miR-370-3p, miR-373-3p, miR-142-3p, miR-210-3p, miR-26a-5p, miR-26b-5p, miR-199a-3p, miR-24-3p, miR-222-3p, miR-19a-3p, miR-17-5p, miR-411-5p, miR-27a-3p, miR-152-3p, miR-29a-3p. Cartilage-destructive: miR-381-3p, miR-100-5p, miR-139-5p, miR-203a-3p, miR-34a-5p, miR-30b-5p, miR-302b-3p, miR-181a-5p, miR-18a-5p, miR-23a-3p, miR-216b-5p, miR-138-5p, miR-101-3p, miR-16-5p, miR-21-5p, miR-223-3p, miR-155-5p, miR-483-5p, and miR-19b-3p, miR-125b-5p.

### 2.7. Total RNA Isolation and miRNA Profiling

miRNeasy and RNeasy Cleanup Kits (Qiagen, Hilden, Germany) were used to extract total RNA from similar iASC-EV numbers (26 ± 4 E9, mean ± SEM), following the manufacturer’s instruction. To monitor the process of RNA recovery and cDNA synthesis, before RNA extraction samples were spiked-in with exogenous ath-miR-159a (30 pg), an Arabidopsis thaliana synthetic miRNA. Specific primers for ath-miR-159a were provided in the RT and PreAmp primer pools (Life Technologies, Foster City, CA, USA). cDNA samples were prepared by standard reverse transcription (RT) and pre-amplification procedures, as previously reported [34]. Expression analysis with the OpenArray system (Life Technologies) was performed into 384-well OpenArray plates, according to the manufacturer’s instructions. An AmpScore value was obtained for each amplification and candidates with AmpScore <1.1 or missing, together with Crt values >25 were considered unamplified. When unamplified, an arbitrary Crt value of 25 was assigned. All assays for RGs and OA-miRNAs were purchased from Life Technologies.

### 2.8. Data Analysis

Efficiency of the whole process was evaluated by scoring the stability of ath-miR-159 Ct values, which were extremely reproducible (18.44 ± 0.12, mean ± SEM) across all samples. Due to the stable values, a spike in ath-miR-159 Crt was used for the initial technical normalization of the other miRNA Crt values before data analysis. Four algorithms were used to calculate RGs stability: geNorm [35], NormFinder [36], BestKeeper [37], and the comparative ΔCt method [38]. Different variables were used by each algorithm to identify given candidates’ expression stability. Linear scale quantitative data is the basis for Normfinder that allows the identification of a stability value that, when low, indicates high stability. geNorm provides an M-value based on the average pairwise expression ratio. Overall, candidates with M < 1.5 are considered as stable. SD is the approach used by BestKeeper to identify best RGs, with a higher SD indicating a less stably expressed RG. The ranking of the RGs according to their stability is generated by each approach, and a series of continuous integers starting from 1 was assigned to each RG. The geometric mean (geomean) of each RG weight across the four programs together with the ranking given by their expression in term of Crt was subsequently determined, leading to a consensus variability score for each reference gene.

Principal Component Analysis (PCA) plots and heatmaps were generated scoring Crt values normalized both with stable miR-26a-5p/16-5p and miR-103a-3p/425-5p with ClustVis package (https://biit.cs.ut.ee/clustvis/) [39]. After row centering, maps were generated using the following settings for both rows and columns clustering distance and method: correlation and average, respectively.

Pearson correlation coefficients were generated between samples, and a post hoc power calculation for linear bivariate regression test was performed using G*power software v3.1.9.2 (Universität Düsseldorf, Düsseldorf, Germany).

Pathway analysis of selected miRNAs was conducted with miRPathDB applet (https://mpd.bioinf.uni-sb.de/overview.html) [40] scoring the Biocarta database (https://www.ebi.ac.uk/miriam/main/datatypes/MIR:00000421) covering several cellular signaling and interaction pathways [41]. The following search parameters were selected: evidence—experimental (strong); minimum number of significant pathways a miRNA should have to be shown—1; and the minimum number of significant miRNAs a pathway should have to be shown—3.

### 2.9. Statistical Analysis

Statistical analyses were performed using GraphPad Prism Software version 5 (GraphPad, San Diego, CA, US). Data were scored for normality by Kolmogorov–Smirnov test. The comparison between the groups was performed using the unpaired Student t-test. Significance level was set at *p*-value ≤ 0.05.

## 3. Results

### 3.1. Characterization of ASCs and EVs

**Figure 1:**
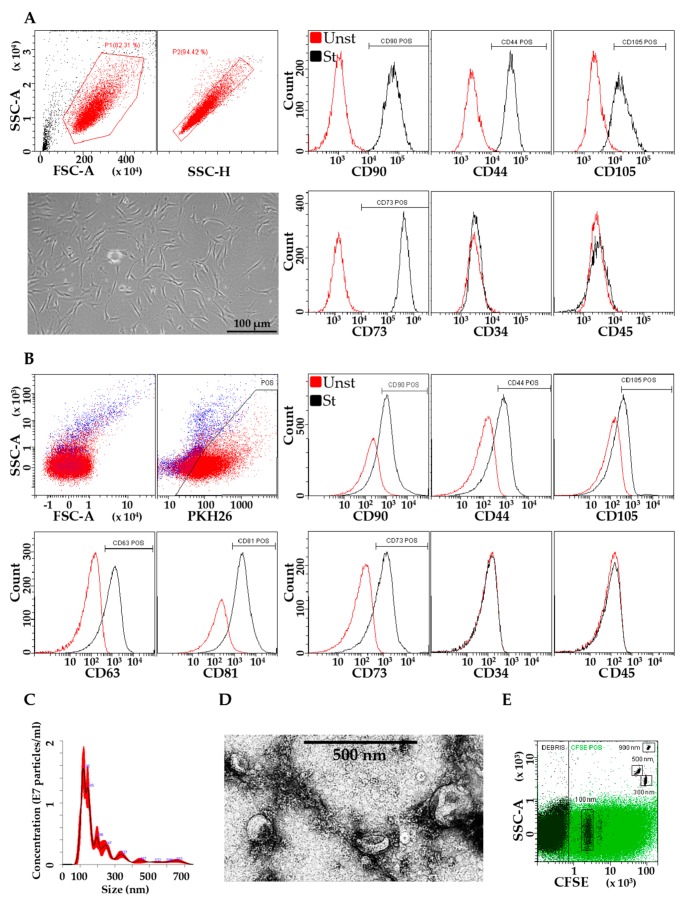
Characterization of ASCs and iASC-EVs. (**A**) Flow cytometry analysis shows the phenotypic resemblance of ASCs being positive to MSC markers (CD44/73/90/105) and negative to hemato/endothelial markers (CD34/45). ASCs also display typical fibroblast-like morphology. Representative cytograms are shown. (**B**) Flow cytometry analysis of iASC-EVs. EVs were stained with PKH26 to allow identification and gating of vesicles in PE channel (in red in the SSC vs. FSC and SSC vs. PE plots). Unstained EVs to set PE gating are shown in blue (SSC vs. FSC and SSC vs. PKH26 plots). After gating, PKH26+ iASC-EVs showed positivity to MSC markers and absence of hemato/endothelial ones. EVs are also positive for extracellular vesicle defining molecules CD63 and CD81. Representative cytograms are presented. (**C**) Size distribution of nanoparticles by NanoSight particle-tracking analysis. (**D**) Transmission electron micrographs of iASC-derived vesicles showing particles with characteristic cup-shaped morphology. (**E**) Cytogram of CFSE-labeled iASC-EVs confirming vesicle integrity.

Flow cytometry of isolated ASCs confirmed the correct MSC phenotype, with fibroblast-like morphology and positive expression of CD44, CD73, CD90, and CD105 markers, in addition to the absence of hemato-endothelial epitopes CD45 and CD34, with its absence/low expression being a fingerprint of expanded ASCs [42] (Figure 1A). After IFNγ stimulation, flow cytometry showed that iASC-EVs expressed both MSC (CD44, CD73, CD90, CD105) and vesicle markers (CD63, CD81), consistent with previously reported characteristics of MSC-EVs (Figure 1B) [22,32,43]. CD34 and CD45 were not detectable. iASC-EVs were inspected by Nanoparticle tracking analysis (NTA) and transmission electron microscopy (TEM). Particles were present in the size of extracellular vesicles (between 50 and 500 nm), with enrichment in small particles (mode size 128 ± 10 nm) within exosomes (Figure 1C). iASC-EVs exhibited the characteristic cup-shape morphology (Figure 1D). Eventually, CFSE staining confirmed that isolated EVs were intact, and—by direct comparison with flow cytometry-dedicated FITC-fluorescent calibration beads of 100, 300, 500, 900 nm—the NTA and TEM estimated size between 50 and 500 nm was confirmed (Figure 1E).

### 3.2. Expression of Candidate Reference miRNAs

**Figure 2:**
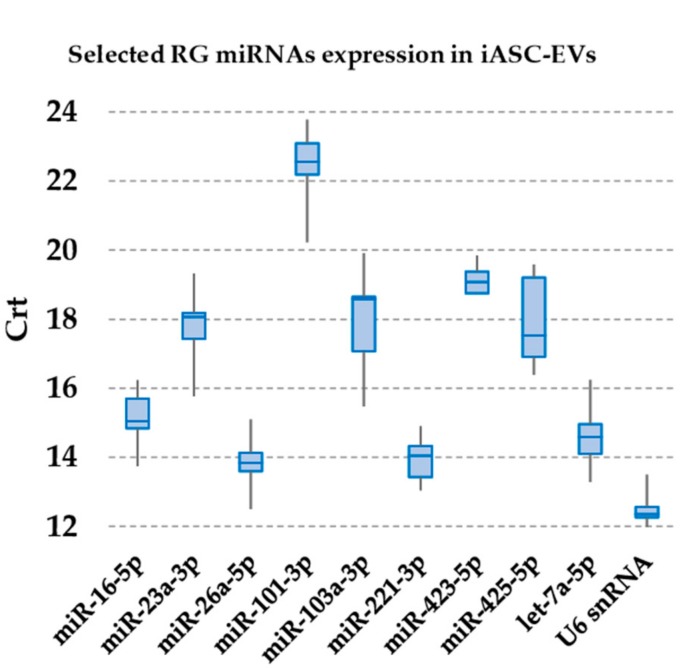
Expression of candidate RG miRNAs in iASC-EVs. The box plot graphs of the Crt values for each RG illustrate the interquartile range (box) and median. The whisker plot depicts the range of the values.

In iASC-EVs samples, U6 snRNA had the highest expression (lowest Crt values), whereas miR-101-3p had the lowest (Figure 2). With the only exception of miR-101-3p, all candidates RG were detected as highly to moderately expressed, with Crt values below 20 in all samples. Moreover, to reduce the likelihood of including co-regulated miRNAs in the study, contiguity analysis evidenced that none of them resides within the same gene cluster [44].

### 3.3. Expression Stability Analysis of RG miRNAs 

Four independent algorithms were used (geNorm, NormFinder, BestKeeper, and the comparative ΔCt method) to rank the stability of the selected RGs in iASC-EVs (Table 1). geNorm analysis identified miR-26a-5p/miR-16-5p (0.228) as the most stable candidates, while miR-425-5p performed poorly (0.741). Notably, all molecules showed an M-value stability lower than 1.5, set by default as the threshold to identify unstable normalizers. NormFinder showed again that miR-16-5p (stability value of 0.060) and miR-26a-5p (0.079) ranked as the best candidates, with miR-103a-3p being the least stable. Using BestKeeper, in iASC-EVs miR-423-5p (0.368) and U6 snRNA (0.401) ranked in the top positions, with reverse ranking for miR-103a-3p (1.332). The comparative ΔCt method results showed similar outcomes to those of the geNorm analysis, with the two most stable RGs being miR-26a-5p (0.536) and miR-16-5p (0.562), while miR-425-5p (1.026) ranked last.

Since the used approaches generated few differences in outcomes, integration and normalization of the data were applied (geomean). Moreover, considering that a good normalizer should be highly expressed, Crt ranking was included in the geomean calculation of each candidate weight. In iASC-EVs, the two most stable RGs were miR-26a-5p (geomean of 1.7) and miR-16-5p (2.2), whereas miR-103a-3p (8.9) and miR-425-5p (9.2) should be avoided.

### 3.4. Assessment of Identified RGs on OA-Related miRNAs

**Figure 3:**
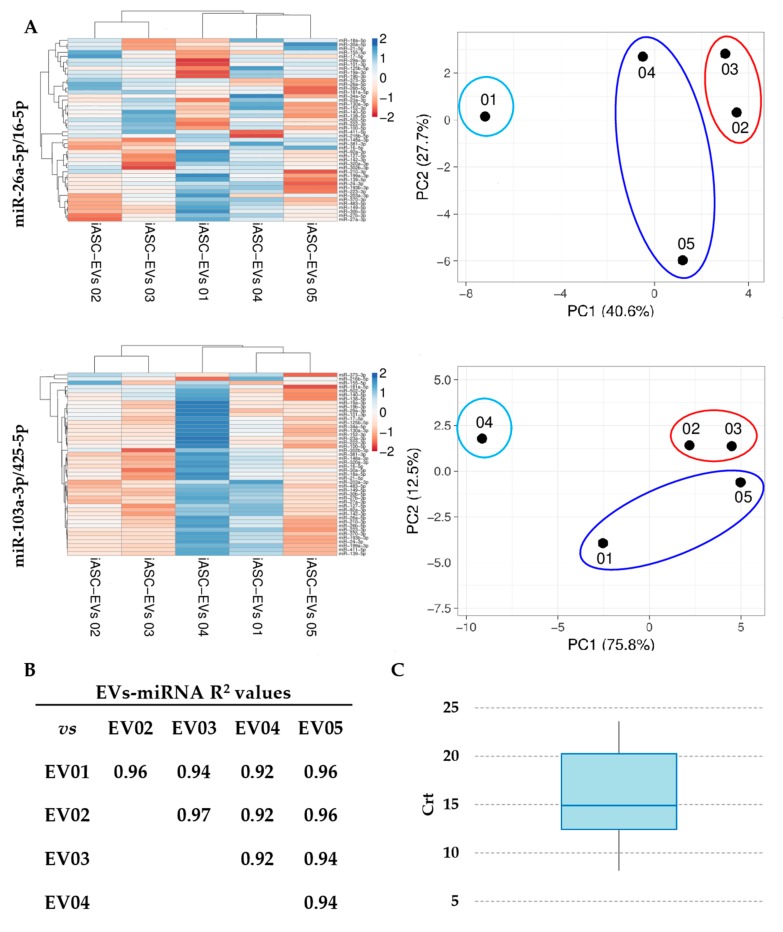
Influence of RG selection on iASC-EVs miRNA profile. (**A**) Heatmap of hierarchical clustering analysis and principal component analysis of the Crt values of 46 iASC-EVs miRNAs after stable miR-26a-5p/16-5p or unreliable miR-103a-3p/425-5p normalization. Rows were centered. Each row represents a miRNA and each column represents a sample. The sample clustering tree is shown at the top. The color scale shown in the map illustrates the relative expression levels of miRNAs across all samples: red shades represent high expression levels (low Crt) and blue shades represent lower expression levels (high Crt). miRNAs from the heat maps are shown in Supplementary Table S2. (**B**) Correlation of miRNA expression levels (normalized Crt) between the five iASC-EVs under study. (**C**) Box-plot of mean normalized Crt values for 46 miRNAs embedded in iASC-EVs.

Forty-six miRNAs involved in cartilage-protective and cartilage-destructive mechanisms in degenerative OA have been previously scored [33]. miR-26a-5p/miR-16-5p and miR-103a-3p/miR-425-5p couples were used to normalize Crt values (Supplementary Table S1 for miR-26a-5p/miR-16-5p) and identify potential differences in the outcomes. Unsupervised clustering analysis was performed to determine whether the expression profile could be used to classify constitutional individuals and to assess the effect of RG choice (Figure 3A). Although in a frame of similarity with the absence of dramatically modulated miRNAs (41 out of 46 miRNAs with SD < 1 and only 2 with SD < 2, miR-373-3p and miR-216b-5p), samples clustered into two groups that were rearranged using the less reliable RGs. In fact, although iASC-EVs 02 and 03 always clustered together, the other three individuals’ hierarchy changed using the miR-103-3p/miR-425-5p combination. In particular, iASC-EV 01 and iASC-EVs 04 inverted their position as leaves of the dendrogram. Moreover, with wrong RGs, iASC-EVs 04 resulted in strong downregulation of all miRNAs (higher Crt values) with respect to the other samples. The same redistribution was also confirmed by principal component analysis that yielded similar results. This finding demonstrated that incorrect RG miRNAs selection may lead to erroneous evaluation of sample interrelation.

**Figure 4:**
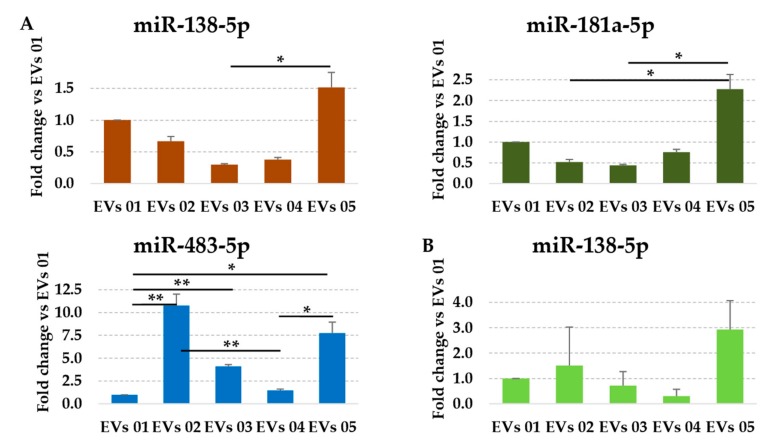
Effects of RGs on the abundance of miRNAs differentially expressed between iASC-EVs samples. (**A**) After normalization with stable miR-26a-5p/16-5p, out of 48 assayed miRNAs only miR-138-5p, miR-181a-5p, and miR-483-5p showed significant differential expression (±4 fold changes) between samples. iASC-EVs 01 set as 1; * *p*-value < 0.05 and ** *p*-value < 0.01. (**B**) Using miR-103a-3p/425-5p on miR-138-5p, both expression ratios (EVs 02 vs. 01 or EVs 03 vs. 04) and significance (EVs 03 vs. 05) are lost.

Since using the most reliable normalization approach a pattern of similarity more than extreme divergence between individuals was observed, a correlation analysis was performed to identify subtle differences (Figure 3B). Notably, R^2^ values higher than 0.9 were always present (power calculation resulted 100% for all comparisons), indicating a conserved pattern of expression for the miRNAs under investigation between the different samples. Then, all expression modulation higher than 4 fold (two normalized Crt values) and statistically significant (*p*-value < 0.05) were scored. To give more strength at the biological significance, miRNAs in the last quartile of expression were excluded (Figure 3C and Supplementary Table S1), due to the recent findings that mesenchymal stem cell-derived EVs may carry no more, and probably even less, than one specific miRNA per particle, even for the most abundant ones [45]. Scoring the remaining 36 miRNAs, only three involved in cartilage destruction (miR-138-5p, miR-181a-5p, and miR-483-5p; Figure 4A) showed significant fluctuations between samples, again suggesting high conservation and homogeneity. Of note, as shown in Figure 4B for miR-138-5p, the use of the wrong combination of RGs generated distorted outcomes, with both erroneous absolute amount evaluation (higher EVs 02 with respect to EVs 01 instead of reduced values) and unreliable ratios (loss of significant difference for EVs 03 vs. 05).

**Figure 5:**
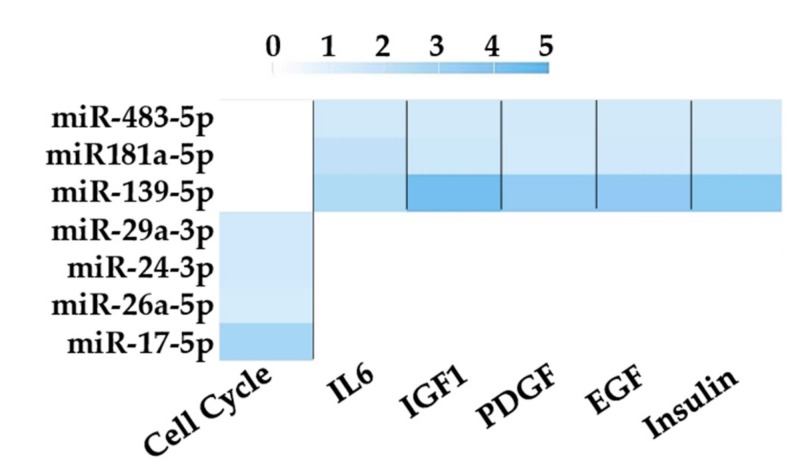
Pathways analysis based on target genes of unique miRNAs using miRPathDB. Enriched pathways (*p*-value < 0.01) regulated by at least three miRNAs and their target genes are shown. Color code bar indicate the number of genes regulated by each specific miRNA for each pathway. Only validated miRNA–gene interactions were selected.

Finally, in silico prediction of other pathways potentially affected by most expressed EV miRNAs was performed using miRPathDB. The database Biocarta was selected and to obtain more stringent results only pathways hit at least by three miRNAs, and their regulated genes, were displayed (Figure 5). Interestingly, six pathways were identified, with five involved in cytokine/chemokine/hormone signaling (IL6, IGF1, PDGF, EGF and Insulin). Notably all these molecules are involved at different levels in regulating cartilage protection or degeneration in OA joints. Interestingly, miR-181a-5p and miR-483-5p through their regulated genes directly modulate all five signaling pathways, suggesting that even few players and fine tuning of their levels, if correctly assessed, may affect target site environment. Eventually, cell cycle—also dysregulated in OA chondrocytes—was the most regulated process with four miRNAs involved. All these data confirm that selected miRNAs may influence joint trophism at different levels.

## 4. Discussion

In this work a rigorous method to identify reliable RG miRNAs in extracellular vesicles derived from adipose derived-MSCs from independent donors has been proposed. miR-26a-5p/16-5p were the best performers, while miR-103a-3p/425-5p behaved poorly, strongly suggesting the use of the former as reliable normalizers for the study of EVs from IFNγ inflamed ASCs. In fact, the selection of appropriate normalizers allowed the identification of subtle differences between donors in OA-related miRNA cargo load, a crucial trait to identify the most suitable product for personalized regenerative medicine approaches.

Mesenchymal stem cell research stands at a critical juncture. There is conflicting evidence as to whether MSCs derived from different healthy donors or, even more critical, from injured or disease patients may have impaired potency [46,47,48,49,50,51,52,53,54,55]. As a pioneeristic report in the field of diseased joints, Murphy and colleagues showed that MSC from OA patients have reduced proliferative and chondrogenic supportive capacity [50]. Moreover, recipients have variable genetic backgrounds, disease types, and/or treatments, all factors influencing treatment success [56]. Therefore, the combination of these elements might in part explain the dichotomy between success of MSC-based efficacy in vitro and in animal models, showing promising but still low outcomes in clinical applications to date [57,58]. To reduce the scenario of multifactorial heterogeneity, it has been recently proposed that MSC pre-activation (cytokines/growth factors and toll like receptors ligands) may generate more homogeneous therapeutic products [10,11,12,18]. Nevertheless, although MSCs may have a comparable and positive response to inflammatory stimuli in terms of secreted factors [59], donor-related differences in cytokine secretion were not completely abolished, as demonstrated for both IL-1β or IFNγ priming [60]. Thus, using a single metric on isolated cultures or derived products as secretome or EVs may be insufficient to predict outcomes, and for this reason disease-specific potency assays have to be developed to select optimal donors for patient specific treatment.

In this frame, with specific focus on MSC-EVs, assays should be focused on shuttled molecules, such as lipids, proteic factors, and small RNAs, such as miRNAs. In fact, EV-associated miRNAs were shown to be crucial regulators of MSC potency [61,62], reinforcing the paradigm that purified EVs may serve as future therapeutic products [63]. Such archetype recently opened the debate for developing off-the-shelf and cell-free MSC therapies in the field of cartilage injuries and osteoarthritis [64]. Therefore, a deep characterization of EVs miRNome is mandatory to both assess the impact of MSC-EVs on target cells/tissues/organs and identify subtle differences between donors. Obtaining both a reliable and validated normalization procedure and selecting stable RGs seems mandatory, and to date candidates are still far to be universally shared. When the amount of RNA is not a limiting factor to score the whole miRNome, a global mean normalization approach is claimed to identify the most stable RG miRNAs within independent samples [44]. In the case RNA amount, as for EVs, and costs are limiting factors, endogenous molecules are preferred [65,66,67]. U6 snRNA was widely used since it was demonstrated to be also present in purified EVs or vesicle-enriched body fluids [68,69]. Nevertheless, just recently U6 snRNA stability was questioned in cardiosphere-derived EVs [23]. Our dataset confirmed that U6 snRNA is not a good performer laying in the central part of the overall stability ranking. This may be due to the fact that U6 snRNA biogenesis is mechanistically separated from miRNA biogenesis [70].

In our experimental settings, miR-26a-5p and miR-16-5p were the best normalizers in iASC-EVs. Interestingly, only miR-16-5p resulted in reliable RGs (second best) in a similar approach performed on ASC-EVs, whereas none emerged as the best housekeeping molecule in EVs isolated from ASCs treated with very low levels of inflammatory cytokines [32]. In the same report, miR-23a-3p was the most reliable in both conditions (resting/inflamed), whereas miR-23a-3p performed poorly. These outcomes suggest that even in similar cell types analyzed by the same laboratory, culture conditions may affect the identification of reliable RGs, and that proper selection must be performed not only for each analyzed cell type but also when external stimuli are added.

Further, proper selection of RGs allowed the comparative analysis of expression levels for miRNAs involved in OA joint trophism [33], and therefore crucial in the view of being transferred in injured tissues. Using unstable miR-103a-3p and miR-425-5p, it clearly emerged as a global over or under-representation of the entire miRNA datasets. In fact, iASC-EVs 04 resulted in all miRNAs being strongly downregulated. Moreover, similarity patterns resulted altered (inversion of iASC-EVs 01 and 04 in the dendrogram), a major issue when comparing biological products for potency prediction. In this frame, although in a pattern of similarity, only proper RG selection allowed the identification of three miRNAs significantly different between samples (miR-138-5p, miR-181a-5p, and miR-483-5p). Interestingly, they are all involved in “cartilage-destructive” mechanisms. miR-138-5p promotes cartilage degradation targeting FOXC1 (Forkhead Box C1) [71], which is involved in endochondral ossification [72]. miR-181a-5p is both pro-inflammatory and pro-catabolic, and targets ZNF440 (zinc finger protein 440), that regulates IκB-independent NF-κB signaling [73]. miR-483-5p stimulates chondrocyte hypertrophy, extracellular matrix (ECM) degradation and cartilage angiogenesis by targeting MATN3 (Matrilin 3) and TIMP2 (Tissue inhibitor of metalloproteinases 2), both involved in initiating and accelerating the development of OA [74]. Therefore, their high expression may indicate a reduced healing capacity for EVs enriched in these factors, as those secreted by iASC 05 in this work, and thus to be evaluated carefully for their selection in OA therapy (Figure 4).

Moreover, in the context of the therapeutic potential of EVs, not only differences between donors have to be taken into consideration, but also the whole message they carry. For embedded miRNAs, the signal given by the most abundant ones may be crucial. In fact, it has been recently proposed that, in MSCs-EVs, fairly one copy of the most abundant miRNAs is embedded [45]. For assayed OA-related miRNAs, considering only the molecules in the first and second quartiles of expression (Supplementary Table S1), a perfect balance between protective and destructive miRNAs is present in the first quartile, whereas in the second one an enrichment of protective moleculescan be found, suggesting a possible overall protective message. Refining our analysis only for the top five expressed miRNAs, miR-24-3p is shown to be involved in downregulating MMP1 and MMP13 expression by targeting senescence marker p16INK4a [75]. miR-125b-5p was shown to promote apoptosis of synovial cells through targeting synoviolin 1 [76]. miR-222-3p may significantly suppress apoptotic death in chondrocytes by down-regulating HDAC4 and MMP13 levels and reducing cartilage destruction [77]. miR-21-5p is upregulated in OA and attenuate the process of chondrogenesis by targeting GDF5 [78]. miR-193b-3p regulates early chondrogenesis and inflammation by inhibiting the TGF-beta2 signaling pathway and MMP19 [79]. Taken together, these results indicate that a number of OA-associated cellular routes may be regulated by transferred miRNAs, rendering strict potency prediction difficult to forecast, also taking into account that other pathways could be potentially influenced (Figure 5). Therefore, small differences, as for miR-138-5p, miR-181a-5p, and miR-483-5p, might get crucial to impact therapeutic efficacy and improve strategies based on the analysis of an array of factors for an effective donor selection process. In the future of multimetric approaches, other components, as miRNAs not already associated with OA pathology and proteic or lipidic factors, must be evaluated with similar validated approaches to score the comprehensive potency of mesenchymal stem cell derived extracellular vesicles. In this frame, a limitation of this study is the solely use of qRT-PCR as assay technique, being aware that other screening approaches, as next generation sequencing, may refine our results from the current study and increase their impact considering the complete miRNome. Furthermore, functional assays on cell types and tissues involved in OA will be needed to score iASC-EVs power as therapeutic products and to establish additional release tests.

## 5. Conclusions

Proper RG miRNA selection is a mandatory process for identifying subtle differences able to predict overall potency of mesenchymal stem cell-derived extracellular vesicles for future use as off-the-shelf therapeutic products in precision medicine. Future characterization of other EV-embedded molecules as other small RNAs (circRNAs, lnRNAs, etc.), proteins, and lipids will be crucial for generating the complete picture of these particles. Establishing proper validation and normalization protocols will be a corner stone for these cell-free based medicines.

## Figures and Tables

**Table 1:** Stability levels of candidate RGs.

Gene name	GeNorm M-Value	NormFinder Stability	BestKeeper SD ± CP	ΔCt Mean	Mean Crt	GeoMean
miR-26a-5p	0.228 (1)	0.079 (2)	0.626 (4)	0.536 (1)	13.81 (2)	1.7
miR-16-5p	0.228 (1)	0.060 (1)	0.683 (5)	0.562 (2)	15.09 (5)	2.2
221-3p	0.313 (3)	0.282 (4)	0.571 (3)	0.648 (3)	13.94 (3)	3.2
U6 snRNA	0.414 (4)	0.423 (7)	0.401 (2)	0.757 (7)	12.53 (1)	3.3
let-7a-5p	0.497 (6)	0.240 (3)	0.773 (6)	0.663 (4)	14.61 (4)	4.4
miR-423-5p	0.439 (5)	0.511 (8)	0.368 (1)	0.834 (8)	19.15 (9)	4.9
miR-23a-3p	0.548 (7)	0.336 (6)	0.926 (8)	0.716 (6)	17.72 (6)	6.6
miR-101-3p	0.583 (8)	0.323 (5)	0.926 (7)	0.702 (5)	22.33 (10)	6.7
miR-103a-3p	0.674 (9)	0.622 (10)	1.332 (10)	0.997 (9)	17.87 (7)	8.9
miR-425-5p	0.741 (10)	0.620 (9)	1.177 (9)	1.026 (10)	17.88 (8)	9.2

miRNAs were ranked according to gene stability as determined by geomean. The numbers in brackets represent the ranking values, regarded as a recommended final ranking.

## Supplementary Materials

The following are available online at https://www.mdpi.com/2073-4409/8/4/369/s1, Table S1: Crt values of scored miRNAs after miR-26a-5p and miR-16-5p normalization, Table S2: Magnification of miRNA lists as per heat maps in Figure 3A.

## References

[1] Heathman T.R., Nienow A.W., McCall M.J., Coopman K., Kara B., Hewitt C.J. (2015). The translation of cell-based therapies: Clinical landscape and manufacturing challenges. Regen. Med..

[2] Fitzsimmons R.E.B., Mazurek M.S., Soos A., Simmons C.A. (2018). Mesenchymal Stromal/Stem Cells in Regenerative Medicine and Tissue Engineering. Stem Cells Int..

[3] Vizoso F.J., Eiro N., Cid S., Schneider J., Perez-Fernandez R. (2017). Mesenchymal Stem Cell Secretome: Toward Cell-Free Therapeutic Strategies in Regenerative Medicine. Int. J. Mol. Sci..

[4] Caplan A.I. (2017). Mesenchymal Stem Cells: Time to Change the Name!. Stem Cells Transl. Med..

[5] Dumont J., Euwart D., Mei B., Estes S., Kshirsagar R. (2016). Human cell lines for biopharmaceutical manufacturing: History, status, and future perspectives. Crit. Rev. Biotechnol..

[6] Williams D.J., Thomas R.J., Hourd P.C., Chandra A., Ratcliffe E., Liu Y., Rayment E.A., Archer J.R. (2012). Precision manufacturing for clinical-quality regenerative medicines. Philos. Trans. A Math. Phys. Eng. Sci..

[7] Carmen J., Burger S.R., McCaman M., Rowley J.A. (2012). Developing assays to address identity, potency, purity and safety: Cell characterization in cell therapy process development. Regen. Med..

[8] Heathman T.R.J., Rafiq Q.A., Chan A.K.C., Coopman K., Nienow A.W., Kara B., Hewitt C.J. (2016). Characterization of human mesenchymal stem cells from multiple donors and the implications for large scale bioprocess development. Biochem. Engin. J..

[9] Redondo-Castro E., Cunningham C., Miller J., Martuscelli L., Aoulad-Ali S., Rothwell N.J., Kielty C.M., Allan S.M., Pinteaux E. (2017). Interleukin-1 primes human mesenchymal stem cells towards an anti-inflammatory and pro-trophic phenotype in vitro. Stem Cell Res. Ther..

[10] Szabó E., Fajka-Boja R., Kriston-Pál É., Hornung Á., Makra I., Kudlik G., Uher F., Katona R.L., Monostori É., Czibula Á. (2015). Licensing by Inflammatory Cytokines Abolishes Heterogeneity of Immunosuppressive Function of Mesenchymal Stem Cell Population. Stem Cells Dev..

[11] Krampera M. (2011). Mesenchymal stromal cell ‘licensing’: A multistep process. Leukemia.

[12] Polchert D., Sobinsky J., Douglas G., Kidd M., Moadsiri A., Reina E., Genrich K., Mehrotra S., Setty S., Smith B. (2008). IFN-gamma activation of mesenchymal stem cells for treatment and prevention of graft versus host disease. Eur. J. Immunol..

[13] Ruiz M., Cosenza S., Maumus M., Jorgensen C., Noël D. (2016). Therapeutic application of mesenchymal stem cells in osteoarthritis. Expert Opin. Biol. Ther..

[14] Manferdini C.l., Maumus M., Gabusi E., Paolella F., Grassi F., Jorgensen C., Fleury-Cappellesso S., Noël D., Lisignoli G. (2015). Lack of anti-inflammatory and anti-catabolic effects on basal inflamed osteoarthritic chondrocytes or synoviocytes by adipose stem cell-conditioned medium. Osteoarthritis Cartilage.

[15] De Luca P., Kouroupis D., Viganò M., Perucca-Orfei C., Kaplan L., Zagra L., de Girolamo L., Correa D., Colombini A. (2019). Human Diseased Articular Cartilage Contains a Mesenchymal Stem Cell-Like Population of Chondroprogenitors with Strong Immunomodulatory Responses. J. Clin. Med..

[16] Crop M.J., Baan C.C., Korevaar S., Ijzermans J.N., Pescatori M., Stubbs A.P., van Ijcken W.F., Dahlke M.H., Eggenhofer E., Weimar W. (2010). Inflammatory conditions affect gene expression and function of human adipose tissue-derived mesenchymal stem cells. Clin. Exp. Immunol..

[17] Maumus M., Roussignol G., Toupet K., Penarier G., Bentz I., Teixeira S., Oustric D., Jung M., Lepage O., Steinberg R. (2016). Utility of a Mouse Model of Osteoarthritis to Demonstrate Cartilage Protection by IFNγ-Primed Equine Mesenchymal Stem Cells. Front. Immunol..

[18] Serejo T.R.T., Silva-Carvalho A.É., Braga L.D.C.F., Neves F.A.R., Pereira R.W., Carvalho J.L., Saldanha-Araujo F. (2019). Assessment of the Immunosuppressive Potential of INF-γ Licensed Adipose Mesenchymal Stem Cells, Their Secretome and Extracellular Vesicles. Cells.

[19] Lopa S., Colombini A., Stanco D., de Girolamo L., Sansone V., Moretti M. (2014). Donor-matched mesenchymal stem cells from knee infrapatellar and subcutaneous adipose tissue of osteoarthritic donors display differential chondrogenic and osteogenic commitment. Eur. Cell Mater..

[20] Dominici M., Le Blanc K., Mueller I., Slaper-Cortenbach I., Marini F., Krause D., Deans R., Keating A., Prockop Dj., Horwitz E. (2006). Minimal criteria for defining multipotent mesenchymal stromal cells. The International Society for Cellular Therapy position statement. Cytotherapy.

[21] Barilani M., Banfi F., Sironi S., Ragni E., Guillaumin S., Polveraccio F., Rosso L., Moro M., Astori G., Pozzobon M. (2018). Low-affinity Nerve Growth Factor Receptor (CD271) Heterogeneous Expression in Adult and Fetal Mesenchymal Stromal Cells. Sci. Rep..

[22] Ragni E., Banfi F., Barilani M., Cherubini A., Parazzi V., Larghi P., Dolo V., Bollati V., Lazzari L. (2017). Extracellular Vesicle-Shuttled mRNA in Mesenchymal Stem Cell Communication. Stem Cells.

[23] Gouin K., Peck K., Antes T., Johnson J.L., Li C., Vaturi S.D., Middleton R., de Couto G., Walravens A.S., Rodriguez-Borlado L. (2017). A comprehensive method for identification of suitable reference genes in extracellular vesicles. J. Extracell. Vesicles.

[24] Lv C., Yang T. (2018). Effective enrichment of urinary exosomes by polyethylene glycol for RNA detection. Biomed. Res..

[25] Li Y., Zhang L., Liu F., Xiang G., Jiang D., Pu X. (2015). Identification of endogenous controls for analyzing serum exosomal miRNA in patients with hepatitis B or hepatocellular carcinoma. Dis. Markers.

[26] Kennel P.J., Saha A., Maldonado D.A., Givens R., Brunjes D.L., Castillero E., Zhang X., Ji R., Yahi A., George I. (2018). Serum exosomal protein profiling for the non-invasive detection of cardiac allograft rejection. J. Heart Lung Transplant..

[27] Ge Q., Zhou Y., Lu J., Bai Y., Xie X., Lu Z. (2014). miRNA in plasma exosome is stable under different storage conditions. Molecules.

[28] Li Y., Xiang G.M., Liu L.L., Liu C., Liu F., Jiang D.N., Pu X.Y. (2015). Assessment of endogenous reference gene suitability for serum exosomal microRNA expression analysis in liver carcinoma resection studies. Mol. Med. Rep..

[29] Santovito D., De Nardis V., Marcantonio P., Mandolini C., Paganelli C., Vitale E., Buttitta F., Bucci M., Mezzetti A., Consoli A. (2014). Plasma exosome microRNA profiling unravels a new potential modulator of adiponectin pathway in diabetes: Effect of glycemic control. J. Clin. Endocrinol. Metab..

[30] Lange T., Stracke S., Rettig R., Lendeckel U., Kuhn J., Schlüter R., Rippe V., Endlich K., Endlich N. (2017). Identification of miR-16 as an endogenous reference gene for the normalization of urinary exosomal miRNA expression data from CKD patients. PLoS ONE.

[31] Cazzoli R., Buttitta F., di Nicola M., Malatesta S., Marchetti A., Rom W.N., Pass H.I. (2013). microRNAs derived from circulating exosomes as noninvasive biomarkers for screening and diagnosing lung cancer. J. Thorac. Oncol..

[32] Ragni E., Perucca Orfei C., De Luca P., Colombini A., Viganò M., Lugano G., Bollati V., de Girolamo L. (2019). Identification of miRNA Reference Genes in Extracellular Vesicles from Adipose Derived Mesenchymal Stem Cells for Studying Osteoarthritis. Int. J. Mol. Sci..

[33] Endisha H., Rockel J., Jurisica I., Kapoor M. (2018). The complex landscape of microRNAs in articular cartilage: Biology, pathology, and therapeutic targets. JCI Insight.

[34] Cavalleri T., Angelici L., Favero C., Dioni L., Mensi C., Bareggi C., Palleschi A., Rimessi A., Consonni D., Bordini L. (2017). Author information Plasmatic extracellular vesicle microRNAs in malignant pleural mesothelioma and asbestos-exposed subjects suggest a 2-miRNA signature as potential biomarker of disease. PLoS ONE.

[35] Vandesompele J., de Preter K., Pattyn F., Poppe B., van Roy N., De Paepe A., Speleman F. (2002). Accurate normalization of real-time quantitative RT-PCR data by geometric averaging of multiple internal control genes. Genome Biol..

[36] Andersen C.L., Jensen J.L., Ørntoft T.F. (2004). Normalization of real-time quantitative reverse transcription-PCR data: A model-based variance estimation approach to identify genes suited for normalization, applied to bladder and colon cancer data sets. Cancer Res..

[37] Pfaffl M.W., Tichopad A., Prgomet C., Neuvians T.P. (2004). Determination of stable housekeeping genes, differentially regulated target genes and sample integrity: BestKeeper--Excel-based tool using pair-wise correlations. Biotechnol. Lett..

[38] Silver N., Best S., Jiang J., Thein S.L. (2006). Selection of housekeeping genes for gene expression studies in human reticulocytes using real-time PCR. BMC Mol. Biol..

[39] Metsalu T., Vilo J. (2015). ClustVis: A web tool for visualizing clustering of multivariate data using Principal Component Analysis and heatmap. Nucleic Acids Res..

[40] Backes C., Kehl T., Stöckel D., Fehlmann T., Schneider L., Meese E., Lenhof H.P., Keller A. (2017). miRPathDB: A new dictionary on microRNAs and target pathways. Nucleic Acids Res..

[41] Nishimura D. (2001). BioCarta. Biotech Soft. Int. Rep..

[42] Mieczkowska A., Schumacher A., Filipowicz N., Wardowska A., Zieliński M., Madanecki P., Nowicka E., Langa P., Deptuła M., Zieliński J. (2018). Immunophenotyping and transcriptional profiling of in vitro cultured human adipose tissue derived stem cells. Sci. Rep..

[43] Ramos L.T., Sánchez-Abarca L.I., Muntión S., Preciado S., Puig N., López-Ruano G., Hernández-Hernández Á., Redondo A., Ortega R., Rodríguez C. (2016). MSC surface markers (CD44, CD73, and CD90) can identify human MSC-derived extracellular vesicles by conventional flow cytometry. Cell Commun. Signal..

[44] Mestdagh P., van Vlierberghe P., de Weer A., Muth D., Westermann F., Speleman F., Vandesompele J. (2009). A novel and universal method for microRNA RT-qPCR data normalization. Genome Biol..

[45] Toh W.S., Lai R.C., Zhang B., Lim S.K. (2018). MSC exosome works through a protein-based mechanism of action. Biochem. Soc. Trans..

[46] Wang X., Takagawa J., Lam V.C., Haddad D.J., Tobler D.L., Mok P.Y., Zhang Y., Clifford B.T., Pinnamaneni K., Saini S.A. (2011). Donor myocardial infarction impairs the therapeutic potential of bone marrow cells by an interleukin-1-mediated inflammatory response. Sci. Transl. Med..

[47] Sanz-Nogués C., O’Brien T. (2014). MSCs isolated from patients with ischemic vascular disease have normal angiogenic potential. Mol. Ther..

[48] Bocelli-Tyndall C., Bracci L., Spagnoli G., Braccini A., Bouchenaki M., Ceredig R., Pistoia V., Martin I., Tyndall A. (2007). Bone marrow mesenchymal stromal cells (BM-MSCs) from healthy donors and auto-immune disease patients reduce the proliferation of autologous- and allogeneic-stimulated lymphocytes in vitro. Rheumatology.

[49] Bacigalupo A., Valle M., Podestà M., Pitto A., Zocchi E., De Flora A., Pozzi S., Luchetti S., Frassoni F., van Lint M.T. (2005). T-cell suppression mediated by mesenchymal stem cells is deficient in patients with severe aplastic anemia. Exp. Hematol..

[50] Murphy J.M., Dixon K., Beck S., Fabian D., Feldman A., Barry F. (2002). Reduced chondrogenic and adipogenic activity of mesenchymal stem cells from patients with advanced osteoarthritis. Arthritis Rheum..

[51] Del Papa N., Quirici N., Soligo D., Scavullo C., Cortiana M., Borsotti C., Maglione W., Comina D.P., Vitali C., Fraticelli P. (2006). Bone marrow endothelial progenitors are defective in systemic sclerosis. Arthritis Rheum..

[52] Kastrinaki M.C., Sidiropoulos P., Roche S., Ringe J., Lehmann S., Kritikos H., Vlahava V.M., Delorme B., Eliopoulos G.D., Jorgensen C. (2008). Functional, molecular and proteomic characterisation of bone marrow mesenchymal stem cells in rheumatoid arthritis. Ann. Rheum. Dis..

[53] Papadaki H.A., Tsagournisakis M., Mastorodemos V., Pontikoglou C., Damianaki A., Pyrovolaki K., Stamatopoulos K., Fassas A., Plaitakis A., Eliopoulos G.D. (2005). Normal bone marrow hematopoietic stem cell reserves and normal stromal cell function support the use of autologous stem cell transplantation in patients with multiple sclerosis. Bone Marrow Transplant..

[54] Mallam E., Kemp K., Wilkins A., Rice C., Scolding N. (2010). Characterization of in vitro expanded bone marrow-derived mesenchymal stem cells from patients with multiple sclerosis. Mult. Scler..

[55] Mazzanti B., Aldinucci A., Biagioli T., Barilaro A., Urbani S., Dal Pozzo S., Amato M.P., Siracusa G., Crescioli C., Manuelli C. (2008). Differences in mesenchymal stem cell cytokine profiles between MS patients and healthy donors: Implication for assessment of disease activity and treatment. J. Neuroimmunol..

[56] Srijaya T.C., Ramasamy T.S., Kasim N.H. (2014). Advancing stem cell therapy from bench to bedside: Lessons from drug therapies. J. Transl. Med..

[57] Trounson A., McDonald C. (2015). Stem cell therapies in clinical trials: Progress and challenges. Cell Stem Cell.

[58] Galderisi U., Calarco A., Melone M., Peluso G. (2014). Is it possible to improve the success rate of cellular therapies based on mesenchymal stem cells?. J. Stem Cells Res. Rev. Rep..

[59] Zhukareva V., Obrocka M., Houle J.D., Fischer I., Neuhuber B. (2010). Secretion profile of human bone marrow stromal cells: Donor variability and response to inflammatory stimuli. Cytokine.

[60] Gray A., Schloss R.S., Yarmush M. (2016). Donor variability among anti-inflammatory pre-activated mesenchymal stromal cells. Technology.

[61] Phinney D.G., Pittenger M.F. (2017). Concise Review: MSC-Derived Exosomes for Cell-Free Therapy. Stem Cells.

[62] Ferguson S.W., Wang J., Lee C.J., Liu M., Neelamegham S., Canty J.M., Nguyen J. (2018). The microRNA regulatory landscape of MSC-derived exosomes: A systems view. Sci Rep..

[63] Cheng L., Zhang K., Wu S., Cui M., Xu T. (2017). Focus on Mesenchymal Stem Cell-Derived Exosomes: Opportunities and Challenges in Cell-Free Therapy. Stem Cells Int..

[64] Toh W.S., Lai R.C., Hui J.H., Lim S.K. (2017). MSC exosome as a cell-free MSC therapy for cartilage regeneration: Implications for osteoarthritis treatment. Semin. Cell Dev. Biol..

[65] Schwarzenbach H., da Silva A.M., Calin G., Pantel K. (2015). Data Normalization Strategies for MicroRNA Quantification. Clin. Chem..

[66] Meyer S.U., Pfaffl M.W., Ulbrich S.E. (2010). Normalization strategies for microRNA profiling experiments: A ‘normal’ way to a hidden layer of complexity?. Biotechnol. Lett..

[67] Pfaffl M.W. (2001). A new mathematical model for relative quantification in real-time RT-PCR. Nucleic Acids Res..

[68] Gray W.D., French K.M., Ghosh-Choudhary S., Maxwell J.T., Brown M.E., Platt M.O., Searles C.D., Davis M.E. (2015). Identification of therapeutic covariant microRNA clusters in hypoxia-treated cardiac progenitor cell exosomes using systems biology. Circ. Res..

[69] Hayashi T., Lombaert I.M., Hauser B.R., Patel V.N., Hoffman M.P. (2017). Exosomal MicroRNA Transport from Salivary Mesenchyme Regulates Epithelial Progenitor Expansion during Organogenesis. Dev. Cell.

[70] Lee Y., Ahn C., Han J., Choi H., Kim J., Yim J., Lee J., Provost P., Rådmark O., Kim S. (2003). The nuclear RNase III Drosha initiates microRNA processing. Nature.

[71] Yuan Y., Zhang G.Q., Chai W., Ni M., Xu C., Chen J.Y. (2016). Silencing of microRNA-138-5p promotes IL-1β-induced cartilage degradation in human chondrocytes by targeting FOXC1: miR-138 promotes cartilage degradation. Bone Joint Res..

[72] Yoshida M., Hata K., Takashima R., Ono K., Nakamura E., Takahata Y., Murakami T., Iseki S., Takano-Yamamoto T., Nishimura R. (2015). The transcription factor Foxc1 is necessary for Ihh-Gli2-regulated endochondral ossification. Nat. Commun..

[73] Nakamura A., Rampersaud Y.R., Sharma A., Lewis S.J., Wu B., Datta P., Sundararajan K., Endisha H., Rossomacha E., Rockel J.S. (2016). Identification of microRNA-181a-5p and microRNA-4454 as mediators of facet cartilage degeneration. JCI Insight.

[74] Wang H., Zhang H., Sun Q., Wang Y., Yang J., Yang J., Zhang T., Luo S., Wang L., Jiang Y. (2017). Intra-articular Delivery of Antago-miR-483-5p Inhibits Osteoarthritis by Modulating Matrilin 3 and Tissue Inhibitor of Metalloproteinase 2. Mol. Ther..

[75] Philipot D., Guérit D., Platano D., Chuchana P., Olivotto E., Espinoza F., Dorandeu A., Pers Y.M., Piette J., Borzi R.M. (2014). p16INK4a and its regulator miR-24 link senescence and chondrocyte terminal differentiation-associated matrix remodeling in osteoarthritis. Arthritis Res. Ther..

[76] Ge F.X., Li H., Yin X. (2017). Upregulation of microRNA-125b-5p is involved in the pathogenesis of osteoarthritis by downregulating SYVN1. Oncol. Rep..

[77] Song J., Jin E.H., Kim D., Kim K.Y., Chun C.H., Jin E.J. (2014). MicroRNA-222 regulates MMP-13 via targeting HDAC-4 during osteoarthritis pathogenesis. BBA Clin..

[78] Zhang Y., Jia J., Yang S., Liu X., Ye S., Tian H. (2014). MicroRNA-21 controls the development of osteoarthritis by targeting GDF-5 in chondrocytes. Exp. Mol. Med..

[79] Chang Z.K., Meng F.G., Zhang Z.Q., Mao G.P., Huang Z.Y., Liao W.M., He A.S. (2018). MicroRNA-193b-3p regulates matrix metalloproteinase 19 expression in interleukin-1β-induced human chondrocytes. J. Cell Biochem..

