# Supplementary material for: Insights into Inflammatory Priming of Adipose-Derived Mesenchymal Stem Cells: Validation of Extracellular Vesicles-Embedded miRNA Reference Genes as A Crucial Step for Donor Selection
Source: Cells. 2019 Apr 23;8(4):369. doi: 10.3390/cells8040369 (PMC6523846; doi:10.3390/cells8040369)
Supplement: Supplementary file 1 [file cells-08-00369-s001.pdf]

Supplementary Table S1. Crt values of scored miRNAs after miR-26a-5p and miR-16-5p normalization.

	iASC-EVs 01	iASC-EVs 02	iASC-EVs 03	iASC-EVs 04	iASC-EVs 05	Mean	Process [33]
miR-24-3p	8.44	8.01	8.28	8.52	7.52	8.15	P
miR-125b-5p	8.01	8.24	8.28	8.57	8.33	8.29	D
miR-222-3p	8.51	9.27	9.69	9.49	8.99	9.19	P
miR-21-5p	9.32	9.61	9.26	9.52	9.77	9.50	D
miR-193b-3p	10.38	9.80	9.85	10.41	9.20	9.93	P
miR-100-5p	10.23	10.91	11.12	10.95	10.32	10.71	D
miR-30b-5p	11.76	11.02	11.09	11.40	11.28	11.31	D
miR-29a-3p	10.30	11.74	11.64	11.69	11.59	11.39	P
miR-92a-3p	12.69	11.41	11.02	11.94	11.21	11.65	P
miR-19b-3p	11.19	12.04	11.72	12.15	11.89	11.80	D
miR-26a-5p	12.23	12.35	12.28	12.13	12.07	12.21	P
miR-34a-5p	12.27	12.24	12.36	13.03	11.93	12.37	D
miR-199a-3p	12.93	12.32	12.44	12.46	12.10	12.45	P
miR-130a-3p	12.33	12.57	12.84	13.03	12.18	12.59	P
miR-30a-5p	12.55	12.85	12.30	12.65	13.32	12.73	P
miR-152-3p	12.77	13.11	13.28	13.56	12.71	13.09	P
miR-210-3p	13.37	13.41	13.09	13.35	12.66	13.17	P
miR-320a-3p	13.30	13.24	13.10	13.29	13.21	13.23	P
miR-17-5p	13.04	13.90	13.35	13.59	13.24	13.42	P
miR-16-5p	13.47	13.35	13.42	13.57	13.63	13.49	D
miR-27a-3p	14.66	13.34	14.02	14.28	14.00	14.06	P
miR-138-5p	13.72	14.31	15.46	15.14	13.13	14.35	D
miR-26b-5p	14.53	14.81	14.62	14.62	14.25	14.57	P
miR-181a-5p	14.40	15.35	15.59	14.81	13.23	14.67	D
miR-27b-3p	15.36	14.30	14.81	15.27	14.77	14.90	P
miR-370-3p	16.02	15.22	15.65	16.02	15.24	15.63	P
miR-483-5p	17.67	14.25	15.63	17.11	14.73	15.88	D
miR-411-5p	15.99	15.93	16.15	15.58	15.86	15.90	P
miR-148a-3p	16.21	15.83	15.67	16.12	16.20	16.00	P
miR-23a-3p	15.48	15.94	16.51	16.60	16.11	16.13	D
miR-19a-3p	16.25	17.24	16.94	17.44	17.18	17.01	P
miR-155-5p	17.18	19.12	18.27	17.52	18.54	18.12	P
miR-149-5p	19.03	17.48	17.73	18.59	18.07	18.18	P
miR-203a-3p	20.83	19.30	20.08	19.56	20.10	19.97	D
miR-18a-5p	19.69	20.18	19.57	20.40	20.09	19.99	D
miR-502-5p	19.42	20.87	21.24	20.29	19.92	20.35	P
miR-140-5p	19.63	20.36	21.74	20.99	19.29	20.40	P
miR-139-5p	20.78	20.44	20.49	20.57	20.18	20.49	D
miR-101-3p	19.94	21.06	20.95	20.71	21.01	20.73	D
miR-373-3p	22.22	22.98	22.46	18.80	17.94	20.88	P
miR-381-3p	21.39	20.38	20.49	22.21	21.53	21.20	D
miR-223-3p	22.53	20.84	20.97	22.07	19.58	21.20	D
miR-216b-5p	25.00	23.79	22.46	17.66	23.21	22.42	D
miR-302b-3p	23.45	23.79	20.21	23.81	22.67	22.79	D
miR-142-3p	24.63	23.30	22.46	23.81	23.21	23.48	P
miR-127-5p	24.82	23.79	22.46	23.81	23.21	23.62	P

Color code represents quartiles of expression. P stands for cartilage-protective mechanisms and D for cartilage-destructive mechanisms involvement.

Supplementary Table S2. Magnification of miRNA lists as per heat maps in Figure 3A.

miR-26a-5p/16-5p	miR-103a-3p/425-5p
miR-18a-5p	miR-373-3p
miR-30a-5p	miR-216b-5p
miR-21-5p	miR-155-5p
miR-155-5p	miR-181a-5p
miR-17-5p	miR-502-5p
miR-29a-3p	miR-140-5p
miR-101-3p	miR-138-5p
miR-125b-5p	miR-19a-3p
miR-19a-3p	miR-19b-3p
miR-19b-3p	miR-29a-3p
miR-373-3p	miR-101-3p
miR-26a-5p	miR-17-5p
miR-26b-5p	miR-125b-5p
miR-181a-5p	miR-34a-5p
miR-34a-5p	miR-130a-3p
miR-23a-3p	miR-152-3p
miR-130a-3p	miR-23a-3p
miR-152-3p	miR-222-3p
miR-140-5p	miR-100-5p
miR-138-5p	miR-302b-3p
miR-502-5p	miR-381-3p
miR-222-3p	miR-148a-3p
miR-100-5p	miR-320a-3p
miR-411-5p	miR-16-5p
miR-216b-5p	miR-30a-5p
miR-148a-3p	miR-18a-5p
miR-381-3p	miR-21-5p
miR-16-5p	miR-203a-3p
miR-92a-3p	miR-483-5p
miR-127-5p	miR-149-5p
miR-142-3p	miR-30b-5p
miR-320a-3p	miR-27b-3p
miR-302b-3p	miR-27a-3p
miR-210-3p	miR-127-5p
miR-199a-3p	miR-92a-3p
miR-139-5p	miR-142-3p
miR-24-3p	miR-26a-5p
miR-193b-3p	miR-210-3p
miR-223-3p	miR-26b-5p
miR-203a-3p	miR-223-3p
miR-370-3p	miR-370-3p
miR-483-5p	miR-193b-3p
miR-149-5p	miR-24-3p
miR-30b-5p	miR-199a-3p
miR-27b-3p	miR-411-5p
miR-27a-3p	miR-139-5p
